# Adaptation, spread and transmission of SARS-CoV-2 in farmed minks and associated humans in the Netherlands

**DOI:** 10.1038/s41467-021-27096-9

**Published:** 2021-11-23

**Authors:** Lu Lu, Reina S. Sikkema, Francisca C. Velkers, David F. Nieuwenhuijse, Egil A. J. Fischer, Paola A. Meijer, Noortje Bouwmeester-Vincken, Ariene Rietveld, Marjolijn C. A. Wegdam-Blans, Paulien Tolsma, Marco Koppelman, Lidwien A. M. Smit, Renate W. Hakze-van der Honing, Wim H. M. van der Poel, Arco N. van der Spek, Marcel A. H. Spierenburg, Robert Jan Molenaar, Jan de Rond, Marieke Augustijn, Mark Woolhouse, J. Arjan Stegeman, Samantha Lycett, Bas B. Oude Munnink, Marion P. G. Koopmans

**Affiliations:** 1 Usher Institute, University of Edinburgh, Edinburgh, UK; 2 Erasmus MC, Department of Viroscience, WHO Collaborating Centre, Rotterdam, the Netherlands; 3 Department Population Health Sciences, Faculty of Veterinary Medicine, Utrecht University, Utrecht, the Netherlands; 4 Municipal Health Service GGD Limburg-Noord, Venlo, the Netherlands; 5 Municipal Health Service GGD Hart voor Brabant, Tilburg, the Netherlands; 6 Stichting PAMM, Veldhoven, the Netherlands; 7 Municipal Health Service GGD Brabant-Zuidoost, Eindhoven, the Netherlands; 8 Sanquin Blood Supply Foundation, Amsterdam, the Netherlands; 9 Institute for Risk Assessment Sciences (IRAS), Utrecht University, Utrecht, the Netherlands; 10 Wageningen Bioveterinary Research, Lelystad, the Netherlands; 11 Netherlands Food and Consumer Product Safety Authority (NVWA), Utrecht, the Netherlands; 12 GD Animal Health, Deventer, the Netherlands; 13 Roslin Institute, University of Edinburgh, Edinburgh, UK

**Keywords:** Viral epidemiology, Molecular evolution, Pathogens, SARS-CoV-2, Epidemiology

## Abstract

In the first wave of the COVID-19 pandemic (April 2020), SARS-CoV-2 was detected in farmed minks and genomic sequencing was performed on mink farms and farm personnel. Here, we describe the outbreak and use sequence data with Bayesian phylodynamic methods to explore SARS-CoV-2 transmission in minks and humans on farms. High number of farm infections (68/126) in minks and farm workers (>50% of farms) were detected, with limited community spread. Three of five initial introductions of SARS-CoV-2 led to subsequent spread between mink farms until November 2020. Viruses belonging to the largest cluster acquired an amino acid substitution in the receptor binding domain of the Spike protein (position 486), evolved faster and spread longer and more widely. Movement of people and distance between farms were statistically significant predictors of virus dispersal between farms. Our study provides novel insights into SARS-CoV-2 transmission between mink farms and highlights the importance of combining genetic information with epidemiological information when investigating outbreaks at the animal-human interface.

## Abstract (web summary)

SARS-CoV-2 was detected in mink farms in the Netherlands in the first wave of the pandemic with evidence of human-to-mink and mink-to-human transmission. Here, the authors investigate this outbreak using phylodynamic analysis and show that personnel links and spatial proximity are predictors of transmission between farms.

## Introduction

Since the initial cluster of cases reported in Wuhan, China, SARS-CoV-2 is predominantly transmitted between people, with occasional examples of transmission between humans and animals. An expanding range of animals has been found to be susceptible and natural infections have been documented particularly in carnivores, including dogs, domesticated cats, lions and tigers, otters and ferrets, which were in contact with infected humans^1,2^. Infections have not been detected in most common livestock species, but multiple countries have reported SARS-CoV-2 in farmed minks to the World Organisation for Animal Health (OIE) (https://wahis.oie.int/#/dashboards/country-or-disease-dashboard).

In the Netherlands, SARS-CoV-2 was first detected in farmed minks in late-April with signs of respiratory symptoms and increased mortality^3^. An in-depth One Health investigation, combining whole-genome sequencing (WGS) with epidemiological information, was conducted in response to the outbreaks in mink farms. The findings of the initial investigation between April and June highlighted that mink sequences from the first 16 farms grouped into five different clusters reflecting independent introductions of the virus from humans to farmed mink. Based on these genetic signatures, it was also shown that people working on the farms were infected with mink strains rather than strains circulating among humans in the same community, providing evidence of animal to human transmission of SARS-CoV-2 at mink farms^4^. Three of the five different clusters continued spreading and in total 68 out of 126 mink farms in the Netherlands were diagnosed with SARS-CoV-2 infections between April and November 2020. From January 2021 onwards all fur farming was banned in the Netherlands. To date, the modes and mechanisms of most farm-to-farm transmissions have remained unknown. Phylodynamic analyses of whole-genome viral sequences from mink farms and associated human cases combined with epidemiological data can help to address specific epidemiological and outbreak-control questions.

In this study, we describe an in-depth molecular epidemiological analysis of the outbreak in 68 mink farms in the Netherlands, as well as humans living and/or working on these mink farms. We used Bayesian phylodynamic methods to gain more insight into the timing of SARS-CoV-2 introductions and the patterns of farm-to-farm transmission. Specifically, we explored the approximate time of onset for the different mink farm clusters and we compared the rate of evolution and population dynamics between mink clusters with the rate of evolution in the human population. Further, we have quantified the virus transmission patterns between different farms and identified farms which are more likely to be the donors of such transmissions; finally, we tried to infer the possible predictors that may drive the transmissions between farms.

## Results

### SARS-CoV-2 infections in mink farms in the Netherlands

**Fig. 1:**
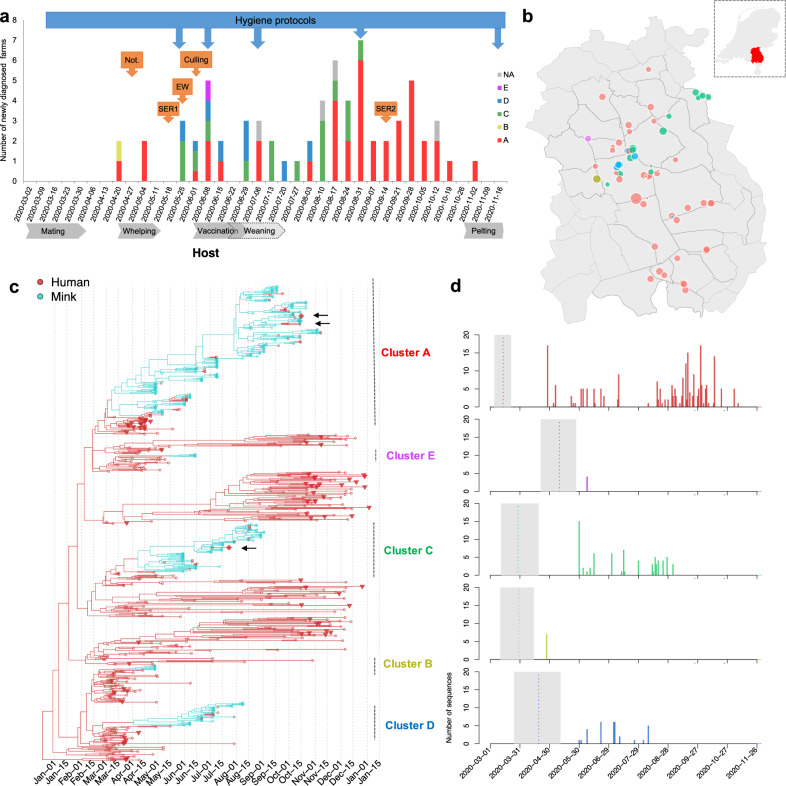
Distinct Clusters of SARS-Cov-2 circulating in mink farms in the Netherlands. **a** Overview of SARS-CoV-2 outbreaks on mink farms in the Netherlands in relation to implementation of control measures and the mink farm cycle. The diagnosed farms per week are coloured based on virus cluster. One farm in June 2020 is indicated as half A/half D as both clusters were found. The blue arrows above the graph point to the starting week of implementation of more strict hygiene protocols with regard to people working on or visiting farms. Orange arrows point to the start of other control measures including obligation for notification of clinical signs and mortality (Not.), first and second serological screening (SER1 and 2), early warning system with weekly sending in of carcasses (EW) and culling of infected farms. Below the graph important periods in the farmed mink production cycle are indicated. These include mating (March), whelping (April/May), vaccination (June) and weaning (June and July). Also, the start of the pelting season is shown. The 5 viral clusters are in unique colour (A in red, B in yellow, C in green, D in blue and E in pink) and are consistently used in (**a**–**d**). **b** The location of each infected mink farm. The node size represents the number of sequences obtained from minks in each farm. The locations of farms on the map have been jittered for privacy reasons. **c** Time-scaled maximum clade credibility (MCC) tree of SARS-CoV-2 sequences isolated from humans and minks in the Netherlands (*n* = 673). Human sequences are highlighted in red and mink sequences in green, the subsampled human samples (*n* = 72) isolated from the same four-digit postal code are highlighted as triangle, and 3 samples (1 escaped mink and 2 un-associated human sequences) which fell within mink clusters are highlighted as diamond and indicated by arrows. Clusters of sequences from minks and associated humans are indicated on the right with unique colours. **d** The number of samples in time for each cluster. The estimated TMRCAs of each cluster are indicated via dotted line (mean) and grey shade (95% highest posterior density (HPD) intervals).

In total minks from 68   of 126 mink farms (farm IDs: NB1 to NB68) in the Netherlands were diagnosed with SARS-CoV-2 between 24 April and 4 November, and these farms were culled within 0–6 days (mean 2, median 1) after sampling from NB8 onwards (Fig. 1a, b). Control measures were implemented immediately after the first infected farms were detected and included culling of infected farms from June onwards. All mink farms were subjected to a ban on transport of animals, animal waste and products, and visitors. Strict hygiene protocols and animal surveillance programmes for early detection were implemented (Fig. 1a).

Most SARS-CoV-2-positive farms were located in a mink farm dense area in the south-east of the Netherlands with 43 farms positive in the province North Brabant and 23 farms positive in Limburg (Fig. 1b). Two farms were located in the province Gelderland, bordering another mink farm dense area. Up to July, on average 1.73 farms (median 1) were diagnosed per week. Despite implemented control measures, and a reduction of activities involving handling of the minks and employing additional staff after the weaning period in July, the weekly number of farms diagnosed increased in August and September to 3.89 (median 3.5), after which it declined to 1.17 (median 1) in October and November (Fig. 1b).

At 41/68 mink farms, employees were confirmed SARS-CoV-2 positive by RT-PCR. Sequences belonging to all five mink clusters were identified from these human samples, the percentage farms with infected humans varying from 55% of farms in Cluster A (22/40) to 100% in Cluster B and Cluster E (1/1). On 31 out of 41 farms, the sampling date of the human positives was after the date of first detection of SARS-CoV-2 in the minks, while for two farms the human sampling dates were unknown. In three out of eight farms, workers tested positive over 1 week before their animals were reported to be SARS-CoV-2 positive.

Between 24 April 2020 and 4 November 2020, we have obtained full viral-genome sequences from 295 minks from 64 out of the 68 infected mink farms (Fig. 1a–c and Supplementary Data 1); no genomes were available from four farms (NB22, NB30, NB37 and NB66). From 57 out of 102 human positives directly linked to 27 farms, a full viral-genome sequence was obtained (Fig. 1c and Supplementary Data 1).

### Introductions and ongoing spreading clusters in mink farms

To look at the transmission of SARS-CoV-2 in mink farms in the Netherlands, we included full-length genomes of SARS-CoV-2 from humans and animals infected on mink farms, and representative SARS-CoV-2 genomes from COVID-19 cases from the general human population of the Netherlands (*n* = 673) to perform a time resolved phylogeographic analysis using BEAST (Fig. 1c). The five distinct mink farm sequence clusters (A–E) were derived from four lineages B.1.8 (Cluster A), B.11 (Cluster B and D), B.1.22 (Cluster C) and B.1.5 (Cluster E), which have been dominantly circulating in the general human population in the Netherlands according to the Pango-lineage descriptions^5^ (version on 1 April 2021).

The largest cluster found on mink farms is the so-called Cluster A, which contains 195 sequences detected on approximately 60% of the infected mink farms (*n* = 40) across 15 municipalities in three provinces sampled between early-April and November 2020 (Fig. 1b). Cluster C and D have been sampled from fewer farms and circulated for shorter time periods: Cluster C viruses were isolated from 15 mink farms between late-May and early-September while Cluster D viruses were isolated from eight mink farms from late-May until early-August. In comparison, Cluster B and Cluster E have only been identified on one farm (NB2 and NB11, respectively) in the early stage of the epizootic with no subsequent spread. The majority of farms were located within 3 km of each other, but not all neighbouring farms were infected with a virus from the same cluster (Fig. 1b).

Seventeen human SARS-CoV-2 sequences from farm NB1–16 between April and May have been described previously^4^, and here we report another 35 human sequences of mink-farm employees in the period June–November (tips in red in Fig. 1c). All human sequences were part of the mink-associated Cluster A, C and D indicating ongoing transmission between minks and humans within the three clusters. All but one of the human sequences were closely related to the sequences of the minks on the same farm. One human sequence of a Cluster C farm (NB 24) belonged to Cluster D, which could be explained by the fact that this employee assisted in the culling of minks at another farm, where minks were infected with a Cluster D virus.

Interestingly, unique clusters were found on the majority of infected farms, only in one farm two different clusters were found: NB8 (infected by viruses belonging to both Cluster A and D in early-June). It is therefore likely this farm was exposed to two sources of viruses.

We estimated the evolution rates of SARS-CoV-2 in mink populations in the Netherlands by using relaxed clock models, with a mean clock rate of 7.9 × 10^−4^ subst/site/year with 95% highest posterior density (HPD) (7.2 × 10^−4^, 8.4 × 10^−4^). This estimate was derived from sequences from mink farms and subsampled background sequences from humans between March 2020 and January 2021. The approximate times for the ancestral jumps from humans to minks were between mid-March (Cluster A, B and C) and late-April (Cluster D and Cluster E) (Fig. 1d). Three clusters (A, C and D) had ongoing spread to more farms from June to November after the initial investigations of the 16 farms between April and June 2020. The last infected farm was detected on 4 November, after which no new infections were detected (Fig. 1a).

### Spill-over into local community and limited onward transmission

In total, 218 sequences isolated from randomly selected patients from 31 postal codes, in the region of SARS-CoV-2-positive mink farms were obtained in period 4 March 2020 to 4 January 2021, to assess possible spill-over to the local community. In addition, all sequences submitted to GISAID from the Netherlands until 4 January were included in the analysis.

On three separate occasions, a mink-related strain, linked to Clusters A and C (Fig. 1c), was detected. Two out of three patients infected with a mink strain (sampling dates in July and August), lived in a province where no infected minks had been reported, and they did not have direct or indirect contact with the mink farming sector. One patient was found in the regional screening in November but did not report any mink-farm contacts. After November, no human infections with mink strains have been detected (Fig. 1c).

Throat swabs of two escaped minks tested positive for SARS-CoV-2. The two minks were caught 450 and 650 metres away from culled mink farm NB58 and NB59, respectively (8 and 9 days after culling). Genome sequencing was successful for one mink sample, and revealed it belonged to Cluster A (Fig. 1c).

### Specific amino acid changes in the spike protein in multiple mink clusters

**Fig. 2:**
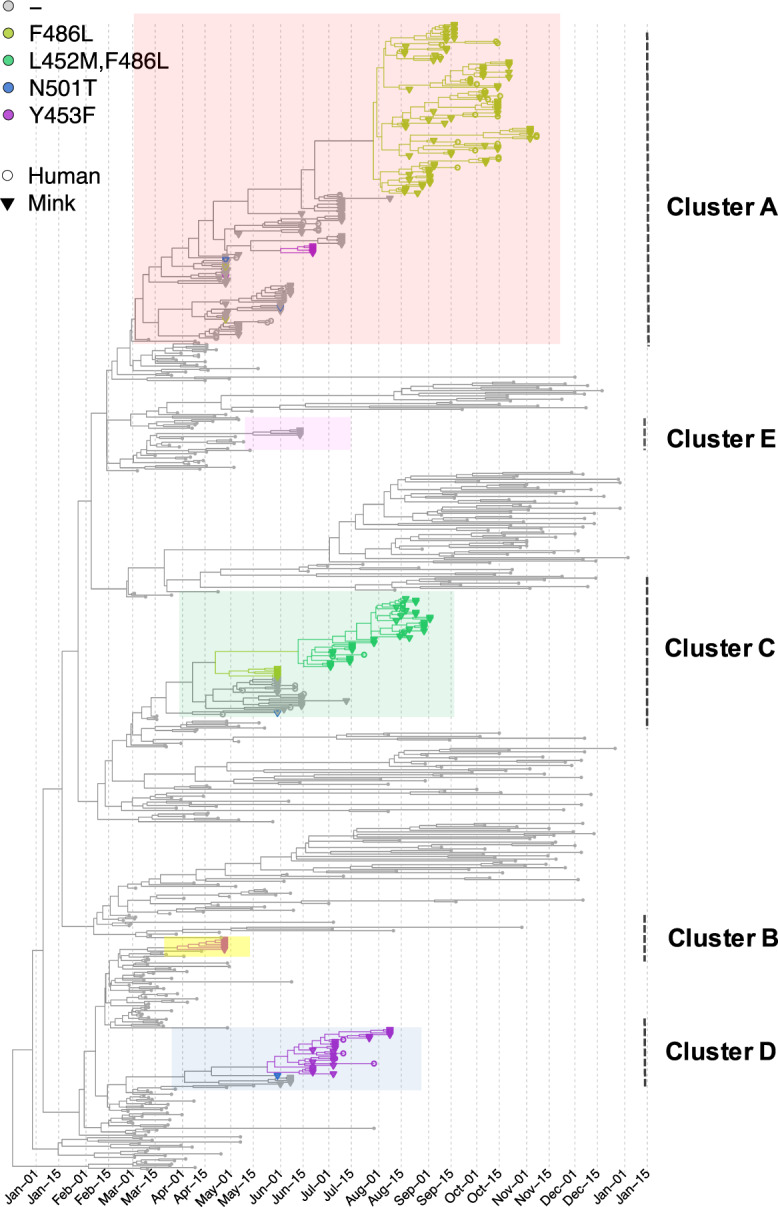
Time-scaled maximum clade credibility (MCC) tree of SARS-Cov2 sequences mapping with 4 amino acid changes **(**L452M, Y453F, F486L and N501T) of the spike protein. The phylogeny is the same as Fig. 1c. Tips with specific amino acid changes are enlarged and in different colours; sequences isolated from mink and human are in triangle and circle, respectively. The 5 viral clusters are highlighted on the tree and labelled on the right.

We further explored how the specific mutations in the spike gene were associated with phylogenies by mapping four potential important amino acid substitutions in the spike protein (L452M, Y453F, F486L, N501T) on the tree composed of the complete dataset (Fig. 2). These four substitutions are in confirmed contact residues of the viral spike protein with the ACE2 receptor^6,7^. Within the Netherlands, these ‘mink-specific’ amino acid changes were only found in minks and employees on mink farms by the time the analysis has been performed (by 1 April 2021), except for three samples: two sequences from the community cases described above (one with F486L, the other with both F486L and L452M) and one sequence from an escaped mink (with F486L). However, these substitutions have also been seen elsewhere in other independent lineages. For example, the F486L has been detected occasionally in humans in Ireland and Columbia, and in mink samples from the US (http://cov-glue.cvr.gla.ac.uk/#/home).

The four substitutions have evolved in multiple clusters and in both human and mink samples from Dutch mink farms. Specifically, F486L substitution has been seen in 217 sequences from 40 mink farms that belong to two separate clusters (A and C), which accounted for 67% of sequences and 68% of sequences isolated within the cluster. Y453F has been seen in 37 sequences from 10 different farms in three different clusters (A, D and E), which accounted for 3%, 82% and 100% of sequences isolated within the cluster. In addition, we found the N501T substitution in four mink virus sequences from three different farms belonging to Cluster A, C and D. L452M was seen in 44 sequences isolated from nine mink farms all belonging to Cluster C (59%). N501T only appeared in a short period of the outbreak (end of April to end of May), while the others appeared in a later stage and sustained longer (F486L first appeared in two sequences in Cluster A at the end of April, then reappeared and replaced F486 in Cluster A since mid-August and in Cluster C since June, respectively); L452M appeared from early-July to September and Y453F appeared from end of April to early-July.

**Fig. 3:**
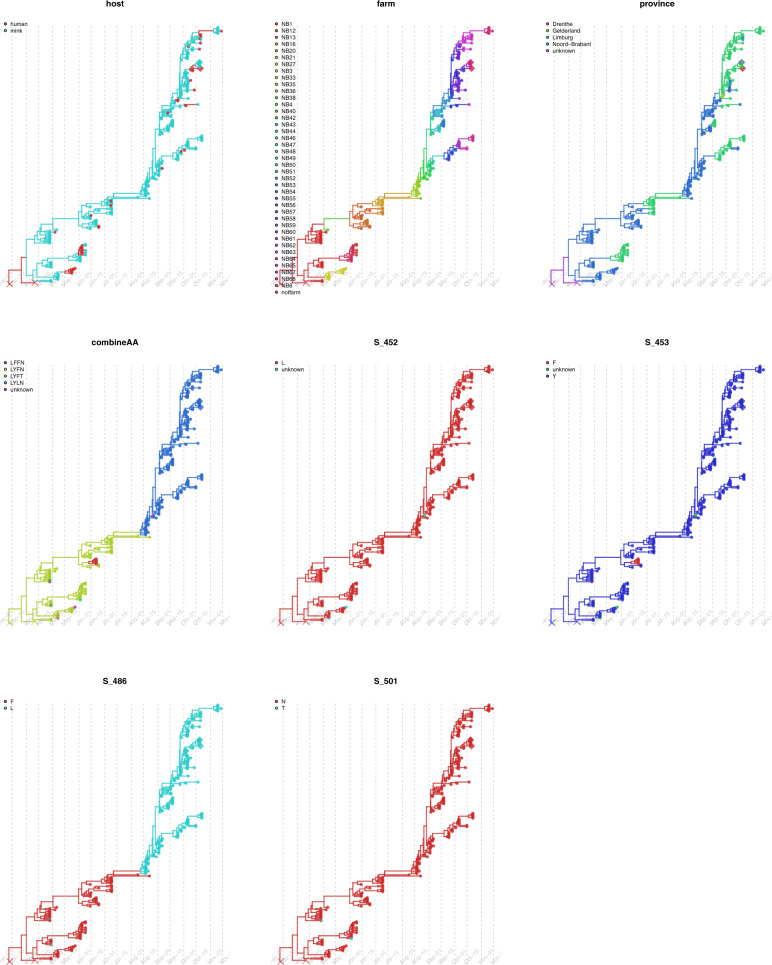
Discrete trait mapping on time-scaled phylogeny of Cluster A. Eight traits including host, farm ID (farm), province, the 4 unique sites in the spike protein with amino acid changes (L452M, Y453F, F486L and N501T) and the combinations of the 4 sites (combineAA) are mapped on the same MCC tree using the discrete trait model. The traits are plotted individually and for each tree, the branches and nodes are coloured by inferred ancestral traits. Samples which fell within mink clusters but not isolated from farms are highlighted in diamond (1 escaped mink and 1 un-associated human sequences). The outgroup containing human samples are cross labelled.

We further mapped the four sites in spike protein with amino acid substitutions (individually and combined), as well as other metadata (host, farm ID and location) on individual time-scaled phylogenies of Cluster A, C and D using discrete trait models. The discrete trait mapping trees of Cluster A are shown in Fig. 3. The trees for Cluster C and Cluster D are shown in Figs. S1 and  S2. The occurrence of the substitutions did not show any significant association to host types, to farm numbers or to locations (Mann-Whitney *U* test, with *p* > 0.5).

In addition, deletions in the spike protein gene resulting in the loss of amino acid residues 141–143 (Del141–143) and residues 141–144 (Del141–144) in the spike protein were identified in two separate mink genomes in the Netherlands. The similar deletions have also been found in five mink sequences in Denmark (four with Del141–143 and one with Del141–144) and in 10 minks from the US (nine with Del141–143 and one with Del141–144).We did not find the deletion in the spike protein gene resulting in the loss of amino acid residues 69 and 70 in the spike protein, which was found in the mink genomes in Denmark and has also been seen on occasions in human variants.

### Comparisons of the phylodynamics of different clusters in minks

**Fig. 4:**
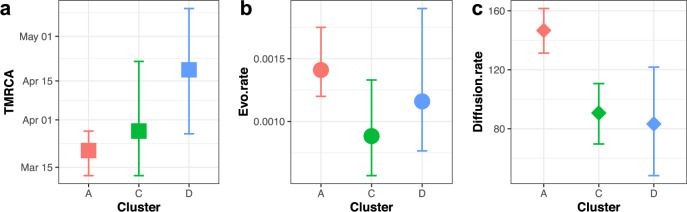
Time to most recent common ancestor (TMRCA), evolution rate and spatial diffusion rate of virus clusters infecting multiple farms. Comparisons of Cluster A, C and D in TMRCA (month, year) (**a**), evolution rate (substitution/site/year) (**b**) and spatial diffusion rate (km/year) (**c**). Data are presented as means (square, circle and diamond) and 95% highest posterior density (HPD) intervals (error bars), estimated with Bayesian phylogenetic methods with MCMC algorithm (with a chain length of 1 × 10^8^ steps sampling every 1 × 10^4^ steps). Cluster A, C and D are in red, green and blue, respectively.

We compared the phylodynamics of three clusters (A, C and D). The results of estimating the time to the most recent common ancestor (TMRCA), the molecular clock evolutionary rate and spatial diffusion rate according to available data and parameters selected are shown in Fig. 4. For Cluster A, the estimated TMRCA for mink sequences is approximately mid-March 2020 (mean 15 March 2020 with 95% HPD (12 March 2020, 28 March 2020); Evolution rate is approximately 1.41 × 10^−3^ subst/site/year with 95% HPD (1.2 × 10^−3^, 1.75 × 10^−3^) subst/site/year. The other two clusters have slightly lower evolution rates and more recent TMRCAs, but with wider HPD intervals; overall these results are consistent with the estimations using a relaxed clock model on the complete data in Fig. 1. The spatial diffusion rate of Cluster A was slightly higher with a mean of 147 km/year (95% HPD, 131–162 km/year) than that of the other two clusters, Cluster C and Cluster D, which have means of 91 km/year (95% HPD, 70–111 km/year) and 83 km/year (95% HPD, 48–122 km/year), respectively (Fig. 4 and Table S1). Overall, the TMRCA for Cluster A sequences aligns with the epidemiological data about the emergence and detection of SARS-CoV-2 in the Netherlands. It also has a faster and wider spatial spread and higher evolutionary rate than the other clusters.

**Fig. 5:**
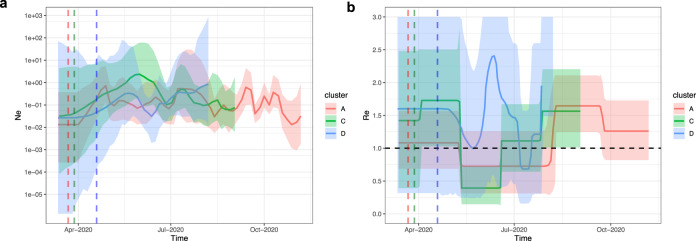
Bayesian Skygrid and BDSKY analysis reveal spatiotemporal independent population dynamics of Cluster A, C and D. **a** Estimation of effective population size (*N*_e_) by Skygrid analysis for Cluster A (red), C (green) and D (blue) sequences. The logarithmic effective number of infections viral generation time (*t*) representing effective transmissions is plotted over time. 95% HPD intervals are plotted in lighter colours. Vertical dashed line is the mean TMRCA. **b** Estimation of reproductive number (*R*_e_) by BDSKY analysis of Cluster A (red), C (green) and D (blue) sequences. The shaded portion is the 95% HPD interval, and the solid line is the posterior median. Vertical dashed line is the mean TMRCA.

We further compared the population dynamics and the transmission potential of the different clusters. The estimated effective population size (*N*_e_) and the estimated reproductive number (*R*_e_) during the course of the outbreak in mink farms are shown in Fig. 5a, b. Here *R*_e_ is representing infection on a between-farm level assuming transmission occurred in a structured population (deme is farm), rather than a between-animal level given the limited number of sequences sampled and smaller genetic diversity per farm. Different patterns of *N*_e_ and *R*_e_ were observed for Cluster A overtime, and for Cluster C and D. For the largest, Cluster A, the population size of mink farm sequences experienced an expansion in late-March 2020 and fluctuated later on. *R*_e_ for Cluster A stayed above 1 after the start of infections then decreased slightly since May 2020. The rate increased again and peaked at approximately 1.6 with 95% HPD (1.2, 2.1) since early-August 2020 and dropped to 1.3 with 95% HPD (0.8, 1.7) from the end of September untill November 2020. For *N*_e_ of Cluster C, a period of slight increase was observed in mid-June 2020, followed by a decline in size from June to September 2020. *R*_e_ for Cluster C stayed above 1 until May 2020 then decreased sharply (below 1) and increased again and stayed at around 1.5 with 95% HPD (1.0, 2.3) from the end of July 2020. In comparison to Cluster A and C, both *N*_e_ and *R*_e_ for Cluster D have larger uncertainties (wide HPD intervals). These results are in line with the epidemiological data: few farms were infected in July 2020 while there was an increase from August 2020 onwards (Fig. 1a). In addition, the timing of *R*_e_ increases in the later stage coincides with the appearance of clades with amino acid changes on spike protein: F486L (in Clusters A and C); L452M (in Cluster C); Y453F (in Cluster D) (Figs. 2, 3 and  5). We observed similar results by using the multi-type birth–death model which showed a strong increase in the number of infections in clades with amino acid changes rather than clades without amino acid changes (Fig. S3).

### Sources and frequencies of the transmissions between different hosts and farms

Host (humans and minks) and farm number labels were added to the sequences, and the number of transmissions between hosts (asymmetric) and between farms (symmetric) were inferred using discrete traits models on the time resolved trees (Figs. 3,  S1 and S2). To avoid sample size effect on the results, sequences were further subsampled to reduce over-representative sequences from the same farm. For transmissions identified by Markov jumps, we also used Bayesian stochastic search variable selection (BSSVS) to identify only statistically significant pairs (with Bayes Factor >3, the higher the value, the stronger the support). We summarised and compared the network among three clusters, Cluster A, C and D.

Overall, at least 43 zoonotic transmissions (with 95% HPD 34–50) from minks to humans likely occurred in multiple farms (Table S2). Specifically, 27 transmissions of viruses belonging to Cluster A occurred within 13 farms (NB1, NB3, NB8, NB13, NB21, NB52, NB55, NB56, NB57, NB58, NB59, NB63, NB68); 10 transmissions of viruses belonging to Cluster C occurred within seven farms (NB7, NB9, NB14, NB17, NB26, NB29, NB32) and six jumps of viruses belonging to Cluster D occurred within three farms (NB15, NB18 and NB19). However, some human infections may also be due to human-to-human infections, between mink-farm employees or farm-owner family members, which is not included in the model. Therefore, the true number of mink-to-human jumps may be slightly lower.

There are also a few jumps between humans and minks from different farms. For example, within Cluster A, a sequence from humans linked to NB49 are likely transmitted from minks on NB47, although the low number of sequences (there is only one mink sequence obtained in NB49) precludes robust conclusions. We found that viruses may jump back and forth between humans and minks. The sequences sampled from humans in NB8 are likely transmitted to minks in NB12, as shown in the phylogeny of Cluster A (Fig. 3). Epidemiology data indeed shows that the two farms have personnel links, which could be the explanation of this observation (Supplementary Data 2).

**Fig. 6:**
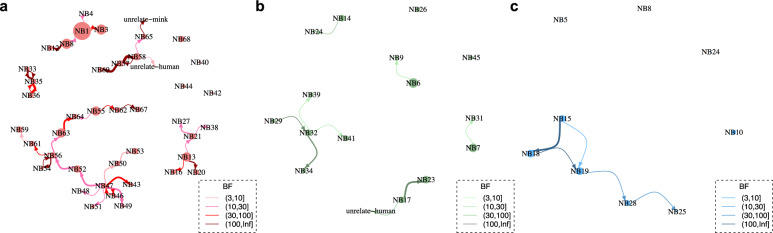
Inferred transmission network between farms. Transmission network between farms inferred from phylogenies of 3 mink clusters (**a**) Cluster A (**b**) Cluster C and (**c**) Cluster D. Size of node indicates number of samples; edge weight indicates median number of transmissions between pairs of farms; arrow on edge indicates transmission direction; colour of edge from light to dark indicates Bayes Factor (BF) support from low to high (only transmissions with BF >3 are shown). The correlated farms are grouped together. Nodes with no link to the others indicated no significant transmissions with other farms although sequences belong to the cluster have been sampled. The number of transmissions and the correlated BF supports are shown in Table S3.

**Fig. 7:**
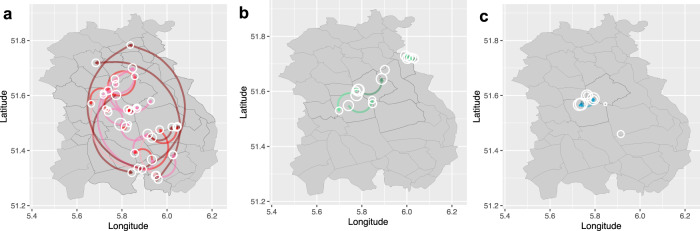
Transmission network between farms on map inferred from phylogenies of 3 mink clusters. **a** Cluster A, **b** Cluster C and **c** Cluster D. The locations of farms on the map have been jittered for privacy reasons. Size of nodes indicates number of samples; arrow on edge indicates transmission direction; colour of edge from light to dark indicates Bayes Factor (BF) support from low to high (only transmissions with BF >3 are shown), colour keys are the same as Fig. 6.

We also identified different potential transmission networks between farms in Cluster A, C and D (Fig. 6 and Table S3). For Cluster A, NB47 seems to be the most important donor, with transmission to seven farms (Fig. 6a). In comparison, fewer significant between-farm transmissions are identified in Cluster C and D (Fig. 6b, c). Transmissions were also drawn as links between locations of mink farms on the map (Fig. 7). Interestingly, we found that transmissions with high Bayes Factor (BF) support (darker red edges in Fig. 7a) are not necessarily between adjacent farms. In addition, sequences from different barns on the same farms do not necessarily group together. For example, within Cluster C, sequences isolated from NB6 at the same date fell into two separate sub-clades.

Assuming the presence of farm-specific signatures allowed linking cases to farms. The two community case sequences are most closely related to sequences from farms NB17 and NB58, respectively; and the sequence from an escaped mink is likely to have a relation with farm NB65 (Fig. 6). However, the patients infected with mink strains did not report any direct or indirect contact with mink or mink-farm employees.

### Inferred predictors of transmissions between farms

During our study, a detailed inventory of possible common characteristics, including farm owner, shared personnel, feed supplier and veterinary service provider was made. Epidemiological investigation indicated that many farms shared the same feed supplier or veterinarian, but no unambiguous service company contacts were found between farms within the different virus clusters which could explain the farm-to-farm spread. For 55% of the SARS-CoV-2-positive farms, owners, family members or personnel, including people with limited contact with minks, were shared between farms (Supplementary Data 2).

Using a generalised linear model (GLM), implemented in BEAST, we tested the contribution of a range of predictor variables to the spread of viruses between farms which was estimated in the discrete trait phylogeographic model (Fig. 6). Correlations between the predictor data collected from mink farms were tested and highly correlated predictors were omitted (Fig. S4). The predictors being tested were: (1) distance between farms; (2) personnel links between farms; (3) feed supplier; (4) veterinary service provider; (5) size of mink population per farm; (6) number of sequences per farm included in the phylogenetic analysis; (7) human population density in municipality where the farm was located; and (8) days between sampling and culling per farm (Supplementary Data 2).

For Cluster A, the distance between farms had a negative impact on the transmission between farms (Table 1), which indicated that farms that are further apart have generally lower rate of transmission between them. Farms with personnel links more often had transmission events, which could be an explanation of the strongly supported long-distance diffusion observed in Fig. 7. For Cluster C and Cluster D, none of the predictors had significant impact on the overall transmission between farms (Table S4).

**Table 1:** The contribution of predictors of SARS-CoV-2 (Cluster A) transmissions between mink farms.

Predictor	Coefficient	95% HPD interval	Inclusion prob.	Coefficient* indicator
Distance between farms^1^*	−0.63	[−0.90, −0.41]	0.99	−0.62
Personnel links^2^*	1.30	[0.51, 2.11]	0.97	1.26
Feed supplier	0.02	[−3.78, 3.85]	0.06	0
Veterinary service provider	0.04	[−3.77, 3.99]	0	0
Mink population of the origin farm	0.004	[−3.95, 3.72]	0	0
Mink population of the destination farm	−0.01	[−3.86, 3.85]	0	0
Sample size of the origin farm	−0.03	[−4.10, 3.78]	0	0
Sample size of the destination farm	0.02	[−3.96, 3.73]	0	0
Human density of the origin farm	−0.11	[−3.81, 3.91]	0.07	−0.01
Human density of the destination farm	0.04	[−3.94, 3.79]	0	0
Days between sampling and culling of origin farm	−0.01	[−3.85, 3.86]	0	0
Days between sampling and culling of destination farm	0.04	[−4.05, 3.76]	0	0

*Predictors included in the model with significant impact.

^1^The shortest distance between farm coordinates estimated in R (package distHaversine).

^2^Links include farms with the same owners, farms sharing employees, farms owned by other members of the same family, or other links like social links and technicians visiting other farms.

## Discussion

In this study, we explored the transmission dynamics of SARS-CoV-2, between mink farms, and between minks and humans, by combining data from SARS-CoV-2 monitoring in humans and animals, associated epidemiological information and by analysing the phylodynamic and transmission patterns of different SARS-CoV-2 sequence clusters in minks and in humans.

SARS-CoV-2 has infected over 200 million people worldwide. Over 1,500,000 genomes had been generated and more than 800 lineages contributed to the active spread globally by 1 April 2021 (when the analysis was performed)^5^. Within the Netherlands, at least 140 lineages have been circulating in humans. We found five distinct clusters (A–E) derived from four different lineages (B.1.8, B.11, B.1.22, B.1.5) which had been dominantly circulating in the general human population in the Netherlands until 1 April 2021. The most recent common ancestors of the five different mink clusters appeared in the Netherlands between mid-March and late-April 2020, which is in line with the timing of initial human detections in the country^8^. The timing of introductions and expansions into mink populations are commensurate with exponential growth of SARS-CoV-2 in the human population in the Netherlands and with the mating season of the farmed minks, which is associated with an increase in external labour with more human-mink contact^3^. The last infected farm was detected in November 2020, after which no new infections were detected, probably due to lack of remaining mink farms with minks in the affected area and the start of the pelting season during which all minks, including the adults, were pelted due to the ban on mink farming from January 2021 onwards.

SARS-CoV-2 infections in minks are concerning as evolution of the virus in an animal reservoir could lead to establishment of additional zoonotic reservoirs with the potential for recurrent spill-over events of novel SARS-CoV-2 variants from minks to humans and other mammals^9^. During its spread and coinciding with a sudden increase in incidence of SARS-CoV-2-positive mink farms in August 2020 we observed that the virus had acquired several amino acid changes compared to the virus last detected at the end of June, with a replacement of circulating viruses with a variant with a F486L substitution in spike protein and some other substitutions also observed in some variants of interest circulating in humans^7,10,11^. It is plausible that the increased phylodynamic growth rate (*R*_e_) after summer 2020 is associated with increased transmissibility in minks due to the emergence of these clades^9,12^. Acquisition of relevant amino acid substitutions was also observed in the Cluster V variant, found in farmed minks in Denmark, which was derived from a Danish-specific lineage B.1.1.298 (https://cov-lineages.org/pango_lineages.html). This variant had three specific amino acid substitutions and one deletion in the spike protein, where Y453F was thought to be strongly associated with the mink infections and may be associated with decreased antibody binding and increased ACE2 affinity^7,10,13^. Cluster V viruses were also found to infect humans and were associated with community transmission after mink-to-human transmission^14^. Currently, viruses with the Y453F substitution have been identified in ~1500 SARS-CoV-2 genomes and in 24 different lineages from Europe, Africa and the USA. Other potentially important substitutions found in minks in Denmark in the spike protein (I692F, M1229I) can also be found in humans globally. Here we found Y453F together with three other substitutions (F486L, 452M and N501T), which were first identified in multiple mink clusters that infected both farmed minks and associated humans in the Netherlands.

We did observe varied phylodynamic and transmission patterns among different mink clusters: the largest Cluster A emerged earlier and has relatively higher evolutionary rate and faster and wider spatial spread over a longer period of time than other clusters. However, in clusters for which we have fewer samples available, we observed higher uncertainty of the estimated phylodynamic parameters (e.g. *N*_e_ and *R*_e_ with wide HPD intervals). Validation of our observations needs to come from laboratory studies with representative viruses. In addition, the possibilities of missing samples in clusters would also lead to a putative bias in the trait analyses and GLM on identifying the significant transmission network and associated predictors and therefore we need to be cautious not to overinterpret the results. For example, the impact of humans on transmission between farms may still be underestimated as it is difficult to identify, locate and sample unregistered or moving workers in mink farms. Moreover, of 102 known human infections on mink farms, only 57 were successfully sequenced, showing that unsampled transmission links may exist. Nonetheless, our data are suggestive of adaptation of viruses to mink, and the evolution of clades with slightly different traits, similar to the variants observed upon circulation among humans. Their exact implications for viral fitness, transmissibility, and antigenicity needs further investigation.

The estimated overall rate (mean of 7.9 × 10^−4^ subst/site/year) of SARS-CoV-2 in minks and humans in the Netherlands is similar to the estimation of the evolution rate of human SARS-CoV-2 indicated in the early stage of the pandemic^15^. The rate of mink Cluster A (mean of 1.41 × 10^−3^ subst/site/year) is more similar to the estimation of the higher rate of human SARS-COV-2 in other studies in China (1.19–1.31 × 10^−3^/site/year)^16^ and worldwide (1.5–3.3 × 10^−3^/site/year)^17^. The evolutionary rate of other human seasonal coronaviruses is fairly similar. It has been shown that the evolutionary rate of the spike protein of coronavirus 299E is around 6.5 × 10^−4^/site/year and for OC43 around 5.7 × 10^−4^/site/year; which are lower compared to for instance influenza A virus, for which the evolution rate is estimated to be around 2.5 × 10^−3^/site/year in the HA gene^18^.

Our trait analysis suggested that a personnel link is one key driver to explain the subsequent transmission among minks and transmission between different mink farms. Other factors than transmissions via humans are less likely to contribute in the cases where long-distance transmissions occurred. Nevertheless, there was generally a positive association with farms in closer proximity, which is consistent with studies on SARS-CoV-2 infections in minks in other countries^19^ and on other pathogens^20–22^. There are also other potential drivers of between-farm transmissions of SARS-CoV-2^23,24^. For example, SARS-CoV-2 has been detected in feral cats and dogs around Dutch mink farms^25,26^. In addition, free-ranging mustelids have tested positive in other countries as well as two escaped mink in our study, and these animals could potentially act as vectors for between-farm transmission^24^. Although for some other pathogens, farm-to-farm transmission via air has been proposed, SARS-CoV-2 RNA in ambient air outside of infected mink farms was not detected^27^. The number of community cases with mink strains around mink farms was nearly absent, making this scenario less likely as well.

Finally, we identified multiple events of mink clusters jumping back and forth between humans and minks within several mink farms. These infections were limited to people associated to the farms with limited spread observed in the general population. However, the mink farming system and associated biosecurity policies may be different in other countries, possibly increasing risk of mink infections for humans. Moreover, with increasing human vaccination rate, as well as potentially animal vaccination, the relative importance and contribution to SARS-CoV-2 evolution of potential animal reservoirs may become more important. Unlike our observations, the Cluster V variant was found in a substantial part of the population in Northern Jutland region of Denmark, although the variant has not been detected anymore after November 2020, potentially due to culling of infected mink farms^14,28^. In all, the findings of the high number of SARS-CoV-2 infections in mink farms and the specific amino acid changes in the spike regions, indicate that continuous surveillance and preventive measures in the fur farming industry^19,29^, as well as other susceptible animal populations are advisable. The emergence of novel variants may also have an effect on the virus’ host range, as has already been shown for the ability to infect mice of the Beta and Gamma variant, as opposed to the wild-type virus and the Alpha variant^30^. Therefore, it is essential to keep monitoring the behaviour of the virus in combination with genetic information in both human and animals, especially animal species that have close contact with humans.

## Methods

### Samples and metadata

#### Mink

Mink farms suspected of SARS-CoV-2 infections were visited for statutory sampling and epidemiological investigation by the competent authority (Netherlands Food and Consumer Product Safety Authority, NVWA). The statutory sampling included non-random sampling using throat and rectal swabs of 20 minks, targeting minks with clinical signs. Farms were visited for statutory sampling based on reporting of increased mortality or respiratory signs by owners or when tested positive during surveillance systems (Fig. 1a). These included an early warning system (EWS) of weekly testing of carcasses of recently dead minks by RT-PCR on nasopharyngeal swabs, with a maximum of five (increased to 50 by the end of August).

Two mandatory serological screenings of all Dutch mink farms (*n* = 60 per farm) were executed end of May and September 2020, by GD Animal Health (GD, Deventer, the Netherlands)^19^.

Throat swabs of two minks, caught at the end of September / beginning of October, 8 and 9 days after culling of two farms (NB58 and NB59) at 450 and 650 m distance, respectively, which most likely escaped during culling, were also submitted for testing. Associated metadata was derived from the database developed by a consortium of One Health outbreak experts. Data collected for each farm included farm location, number of animals, ownership, shared personnel and other contacts (anonymised), and data of confirmed SARS-CoV-2 detection and time interval between sampling and culling. The epidemiology data are in Supplementary Data 2.

#### Human cases associated to mink farms

On the first SARS-CoV-2-infected mink farms, NB1-NB16 (NB is the Dutch abbreviation for mink farm, which were numbered consecutively based on diagnosis) active case finding, as well as serum collection of people with possible exposure to infected minks was performed, as described previously^4^. On farms NB17-NB68, all owners and employees of infected mink farms were requested to visit a regional SARS-CoV-2 testing facility in case of any symptoms indicative of COVID-19, in line with the national SARS-CoV-2 testing and surveillance policy. There were no serum samples taken for antibody detection.

#### Four-digit postal code screening

Two screenings of SARS-CoV-2-positive humans living in the same region as the infected mink farms took place from 3 April 2020 to 16 November 2020. The first screening included a set of sequences obtained from anonymised samples from patients that had been diagnosed with COVID-19 in the area of the same four-digit postal codes as farms NB1-NB4 in March and April 2020, as described previously^4^. For the second screening, municipal health centres selected anonymised laboratory IDs for 10 SARS-CoV-2-positive humans in the period 15 October 2020 to 16 November 2020 from the same postal code regions of the 68 SARS-CoV-2-positive mink farms from their notification system. Based on the laboratory ID, stored samples were retrieved from the diagnostic centres for sequencing. In some regions the number of samples was lower than 10, due to low number of positives in the selected period or because not all samples had been retained by the laboratories. Samples from the selected postal codes that were collected in the period 27 November 2020 to 4 January 2021 were also included in the analysis.

#### SARS-CoV-2 diagnostics and sequencing

Human and animal cases were diagnosed by SARS-CoV-2 RT-PCR testing of oropharyngeal and rectal swabs (minks) or upper respiratory tract samples (humans) in one of the laboratories participating in the national COVID-19 response^31^. RT-PCR-positive samples were processed for WGS as described previously^4^. For each mink farm, a maximum of five of the RT-PCR-positive samples with Ct < 32 were selected, based on lowest Ct values.

For the mandatory serological screening in mink, blood on filter paper was eluated and approximately 2 µL of serum was tested for SARS-CoV-2 antibodies using an in-house indirect ELISA based on the RBD antigen. The same ELISA using the S1 antigen was used for confirmation^32^.

The first and last 30 nucleotides were trimmed, and subsequently mapped against the NC_045512.2 SARS-CoV-2 reference genome using minimap2^33^. After mapping, the alignment files were used to generate a consensus sequence using pysam module^34^ in a custom python script. Homopolymeric regions were manually checked and resolved by consulting reference genomes and positions with less than 30x coverage were replaced with “N”^35^. The complete sequences information and metadata used in the phylogenetic analyses are in Supplementary Data 1.

### Phylodynamic reconstructions

Complete SARS-CoV-2 genomes with >95% coverage isolated from minks and associated humans were included in the phylodynamic reconstructions. We also included human sequences from across the Netherlands as background data. The data were obtained from GISAID (https://www.gisaid.org/) and the collected date was up to 4 January 2021. We then subsampled these background human sequences to keep at least one sequence per global lineage as defined using the Pango-lineage classification (version 1 April 2021)^5^ per region per week.

Genomes were aligned with MAFFT^35^ and edited by partitioning into coding regions and non-coding intergenic regions with a final alignment length of 29,508 nucleotides. Phylogenetic trees were first generated using IQtree^36^ employing maximum likelihood (ML) under 1000 bootstraps. The nucleotide substitution model used for all phylogenetic analyses was HKY with a Gamma rate heterogeneity among sites with four rate categories. To determine if our sequence data exhibited temporal qualities, we used TempEst v1.5^37^ to measure the root-to-tip divergence for ML trees.

Phylodynamic analyses of SARS-CoV-2 in mink farms in the Netherlands were conducted using time-scaled Bayesian phylogenetic methods in BEAST version 1.10.4^38^. The best fit models were HKY + G + 4 for the site substitution model and Skygrid^39^ for the tree model, determined by using stepping-stone sampling^40^. We first generated phylogeny using all full-length genomes of SARS-CoV-2 from mink farms with background human samples using an uncorrelated relaxed molecular clock model which assumes each branch has its own independent substitution rate^41^, We then generated independent phylogenies of Cluster A, C and D using a strict molecular clock model with prior specified (a mean of 1 × 10^−3^ with 95% HPD between 6 × 10^−4^ and 2 × 10^−3^). To analyse fluctuations in SARS-COV-2 epidemic spread in mink farms in the Netherlands per individual cluster, we estimated the changes of viral effective population size (*N*_e_) over time using the Skygrid model^39^ in BEAST version 1.10.4, and the effective reproductive number (*R*_e_) during the course of the outbreak in mink farms, using the birth–death skyline (BDSKY) model^42^ in BEAST2 version 2.6.3^43^. We also used the MultiTypeTree birth–death model (BDMM) to explore whether the appearances of certain amino acid changes in the spike protein have impact on the *R*_e_ variations^44^. We specified the following priors according to the knowledge of SARS-CoV-2 infections in humans and our epidemiology surveillance data on mink farm infections: (1) *R*_e_: a mean of *R*_0_ 2.5 with 95% HPD (0.6, 6)^23,45^, and were estimated over five equidistant time intervals depending on the size of the overall tree; (2) the “becomeUninfectiousRate”, which refers to the number of days from infection to culling for a mink/farm: a mean of 26 (equivalent to 14 days) with 95% HPD between 5 and 20 days; (3) the sampling portion, which refers to the number of sequences per farm divided by the total infected mink population of a farm: a mean of 2 × 10^−4^ with 95% HPD (1 × 10^−5^, 1 × 10^−3^); and (4) the origin time of the epidemic: the estimated TMRCAs of the three mink clusters under strict clock model with priors described above. For each analysis the MCMC algorithm was run for 10^8^ steps and sampled every 10^4^ steps. In addition, we compared the spatial diffusion rates among the three clusters using the coordinates of each infected mink farm via a continuous model^46^ in BEAST version 1.10.4, and validated by scaling the relative rates estimated in the GEO-SPHERE package in BEAST2 version 2.6.3.

We further estimated the transmissions between farms and between minks and humans using the phylogenies of Cluster A, C and D separately. We used an asymmetric model and incorporated BSSVS to identify a sparse set of transmission rates that identify the statistically supported connectivity^47^. We also estimated the expected number of transmissions (jumps) between farms and hosts using Markov rewards^48^. Finally, we inferred the possible predictors that may drive to the spread of virus between farms (estimated between-farm transmission rates) using a GLM, an extension of the discrete diffusion model^49^.

### Medical ethical clearance

Outbreak investigations of notifiable diseases such as COVID-19 are the legal tasks of the Public Health Service as described under the Public Health Act, and do not require separate medical ethical clearance.

### Reporting summary

Further information on research design is available in the Nature Research Reporting Summary linked to this article.

## Supplementary information

Supplementary information.

Reporting summary.

Description of Additional Supplementary Files.

Supplementary Data 1.

Supplementary Data 2.

## Data Availability

Data supporting the findings of this study are available within the article and its Supplementary Information files. All consensus sequences generated in this study have been deposited into GISAID and the sequence reads have been deposited into the European Nucleotide Archive (ENA) (https://www.ebi.ac.uk/ena/browser/home). The accession IDs are listed in the Supplementary Data 1. The source data including metadata of all the sequences and epidemiology data used in all analyses are available in Supplementary Data 1 and 2, respectively.
