# Supplementary material for: Adaptation, spread and transmission of SARS-CoV-2 in farmed minks and associated humans in the Netherlands
Source: Nat Commun. 2021 Nov 23;12:6802. doi: 10.1038/s41467-021-27096-9 (PMC8611045; doi:10.1038/s41467-021-27096-9)
Supplement: Supplementary file 1 — Supplementary information. [file 41467_2021_27096_MOESM1_ESM.pdf]

Supplementary tables and figures

Table S1 Estimated TMRCA, evolution rate and spatial diffusion rate of Cluster A, C and D

Cluster	TMRCA		Last sampling date	Evolution.rate x10-3		Diffusion.rate x10-4	
	mean	95% HPD		mean	95% HPD	mean	95% HPD
A	21/03/2020	[10/03/2020, 10/04/2020]	04/11/2020	1.41	[1.20, 1.75]	2.91	[2.45, 3.38]
C	28/03/2020	[12/03/2020, 22/04/2020]	03/09/2020	0.88	[0.57, 1.04]	1.06	[0.81, 1.34]
D	19/04/2020	[27/03/2020, 11/05/2020]	08/08/2020	1.16	[0.77, 1.60]	1.34	[0.61, 2.2]

Table S2 Number of significant (BF>3) transmissions between human and minks of Cluster A, C and D

Cluster	host	BF2	median_jump	95% HPD interval
A	human->mink	>100	4	(0, 6)
	mink->human	>100	27	(22, 32)
C	human->mink	>100	1	(0, 1)
	mink->human	>100	10	(7, 12)
D	human->mink	>100	0	(0, 1)
	mink->human	>100	6	(5, 6)

Table S3 Number of significant (BF>3) transmissions between farms

Cluster	farm	BF	median_jump	95% HPD interval
A	NB1->NB3	(30,100]	1	(0, 2)
	NB1->NB4	(10,30]	1	(0, 2)
	NB1->NB8	(10,30]	1	(0, 2)
	NB13->NB16	(30,100]	1	(0, 2)
	NB13->NB20	(100,Inf]	2	(1, 4)
	NB13->NB21	(10,30]	1	(0, 2)
	NB21->NB27	(10,30]	1	(0, 2)
	NB21->NB38	(10,30]	1	(0, 2)
	NB33->NB35	(100,Inf]	1	(0, 5)
	NB35->NB33	(100,Inf]	3	(0, 6)
	NB35->NB36	(30,100]	4	(0, 7)
	NB36->NB35	(30,100]	1	(0, 4)
	NB46->NB47	(30,100]	1	(0, 7)
	NB47->NB43	(30,100]	3	(0, 5)
	NB47->NB46	(30,100]	3	(0, 6)
	NB47->NB48	(10,30]	1	(0, 2)
	NB47->NB49	(10,30]	3	(0, 6)
	NB47->NB50	(3,10]	2	(0, 3)
	NB47->NB51	(10,30]	1	(0, 2)
	NB47->NB52	(10,30]	3	(0, 7)
	NB50->NB53	(3,10]	2	(1, 3)
	NB52->NB56	(10,30]	3	(0, 6)
	NB54->NB56	(100,Inf]	1	(0, 5)
	NB55->NB62	(30,100]	1	(0, 2)
	NB55->NB64	(10,30]	1	(0, 2)

	NB56->NB54	(100,Inf]	2		(0, 4)
	NB56->NB61	(30,100]	1		(0, 4)
	NB56->NB63	(10,30]	2		(0, 6)
	NB57->NB58	(100,Inf]	5		(0, 7)
	NB57->NB60	(100,Inf]	4		(0, 6)
	NB58->NB57	(100,Inf]	1		(0, 5)
	NB58->NB65	(10,30]	1		(0, 2)
	NB58->unrelate-human	(3,10]	1		(0, 2)
	NB61->NB59	(3,10]	1		(0, 4)
	NB62->NB67	(100,Inf]	1		(1, 2)
	NB63->NB64	(30,100]	3		(0, 5)
	NB65->unrelate-mink	(100,Inf]	1		(1, 1)
	NB8->NB12	(100,Inf]		3	(1, 5)
C	NB14->NB24	(10,30]	1		(0, 1)
	NB17->NB23	(100,Inf]	3		(1, 5)
	NB17->unrelate-human	(30,100]	1		(0, 1)
	NB7->NB31	(10,30]	1		(0, 1)
	NB29->NB32	(100,Inf]	1		(0, 5)
	NB32->NB34	(100,Inf]	2		(0, 5)
	NB32->NB39	(10,30]	1		(0, 4)
	NB32->NB41	(10,30]	1		(0, 2)
	NB6->NB9	(10,30]	1		(0, 3)
D	NB15->NB18	>100	3		(0, 3)
	NB15->NB19	(3,10]	1		(0, 2)
	NB18->NB19	>100	1		(0, 2)
	NB19->NB25	(30,100]	1		(0, 2)

	NB25->NB28	(30,100]	1	(0, 2)

Table S4 The contribution of predictors of Cluster C and D transmissions between mink farms

Cluster	Predictor	Coefficient	95% HPD interval	Inclusion Prob	Coefficient*Indicator
C	distance between farms	-0.24	[-3.45, 3.67]	0.38	-0.09
	personnel links	0.01	[-3.79, 3.86]	0.04	0
	feed supplier	-0.01	[-4.01, 3.85]	0.01	0
	veterinary service provider	0.02	[-3.855, 3.9941]	0.02	0
	mink population of the origin	0.08	[-4.08, 3.87]	0.03	0.002
	mink population of the destination	-0.01	[-3.84, 3.94]	0.06	-0.001
	sample size of the origin	-0.02	[-3.88, 3.76]	0.01	0
	sample size of the destination	-0.01	[-3.96, 3.88]	0.01	0
	human density of origin	0.03	[-3.93, 3.87]	0.01	0
	human density of destination	-0.002	[-3.89, 3.97]	0.01	0
	days between sampling and culling origin	0.10	[-3.76, 3.88]	0	0
	days between sampling and culling destination	-0.01	[-3.77, 4.08]	0	0
D	distance between farms	0.01	[-3.77, 3.92]	0.01	0
	personnel links	0.02	[-4.04, 3.76]	0.01	0
	feed supplier	0	[-4.01, 3.81]	0	0
	veterinary service provider	-0.02	[-3.72, 3.99]	0	0
	mink population of the origin	0.02	[-3.54, 4.17]	0	0
	mink population of the destination	0.01	[-4.07, 3.75]	0	0
	sample size of the origin	0.20	[-3.86, 4.02]	0.07	0.014
	sample size of the destination	-0.01	[-3.89, 3.74]	0.00	0
	human density of origin	0.12	[-3.78, 4.05]	0.06	0.01

human density of destination	-0.01	[-3.74, 4.03]	0.01	0
days between sampling and culling origin	0.03	[-3.90, 3.82]	0.01	0
days between sampling and culling destination	-0.01	[-3.89, 3.80]	0	0

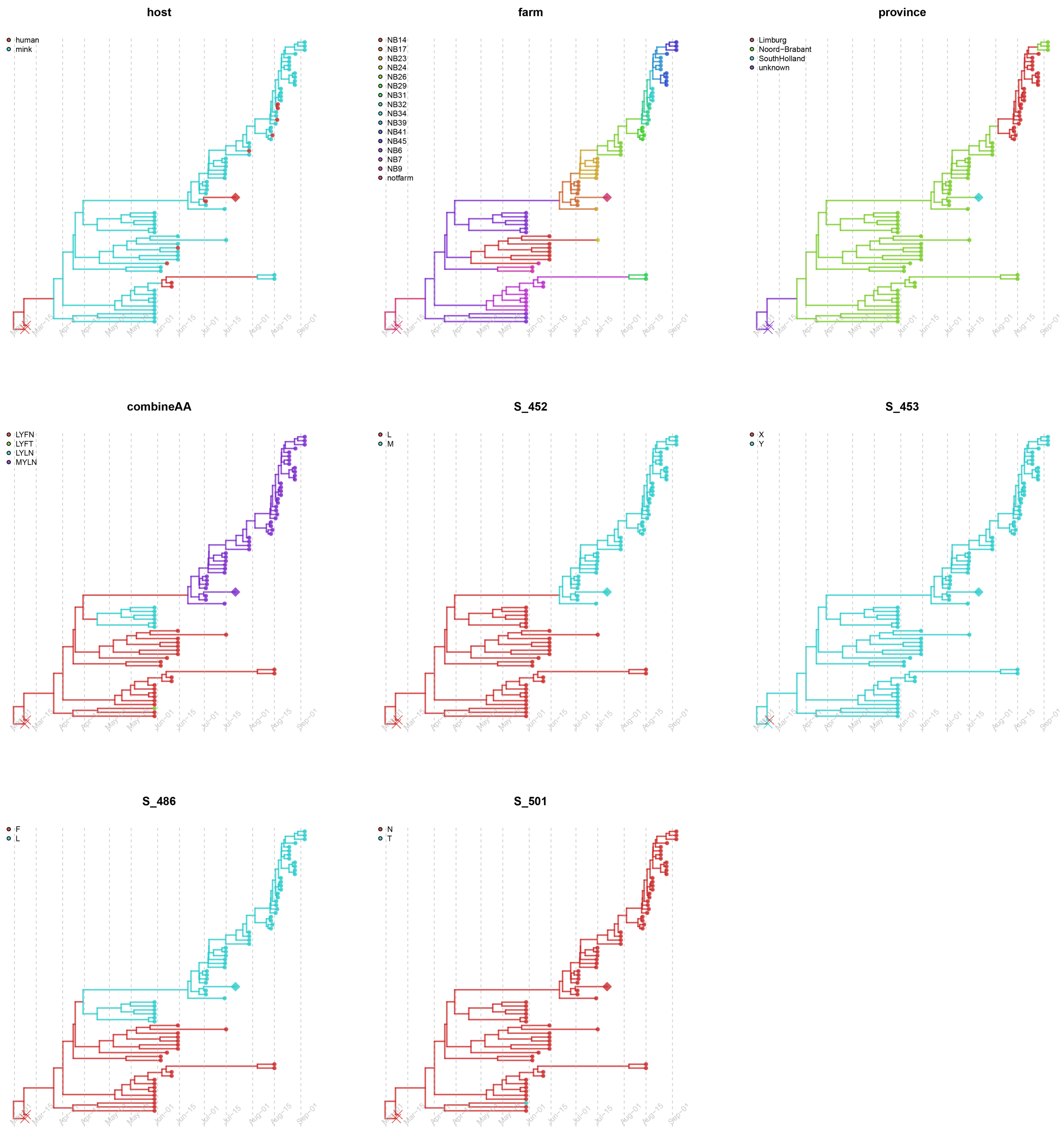

Figure S1. Discrete trait mapping on time-scaled phylogeny of Cluster C. Eight traits including host, farm ID (farm), province, the 4 unique sites in the spike protein with amino acid changes (L452M, Y453F,F486L and N501T) and the combinations of the 4 sites (combineAA) are mapped on the same MCC tree using the discrete trait model. The traits are plotted individually and for each tree, the branches and nodes are colored by inferred ancestral traits. Samples which fell within mink clusters but not isolated from farms are highlighted in diamond (1 escaped mink and 1un-associated human sequences). The outgroup containing human samples are cross labelled.

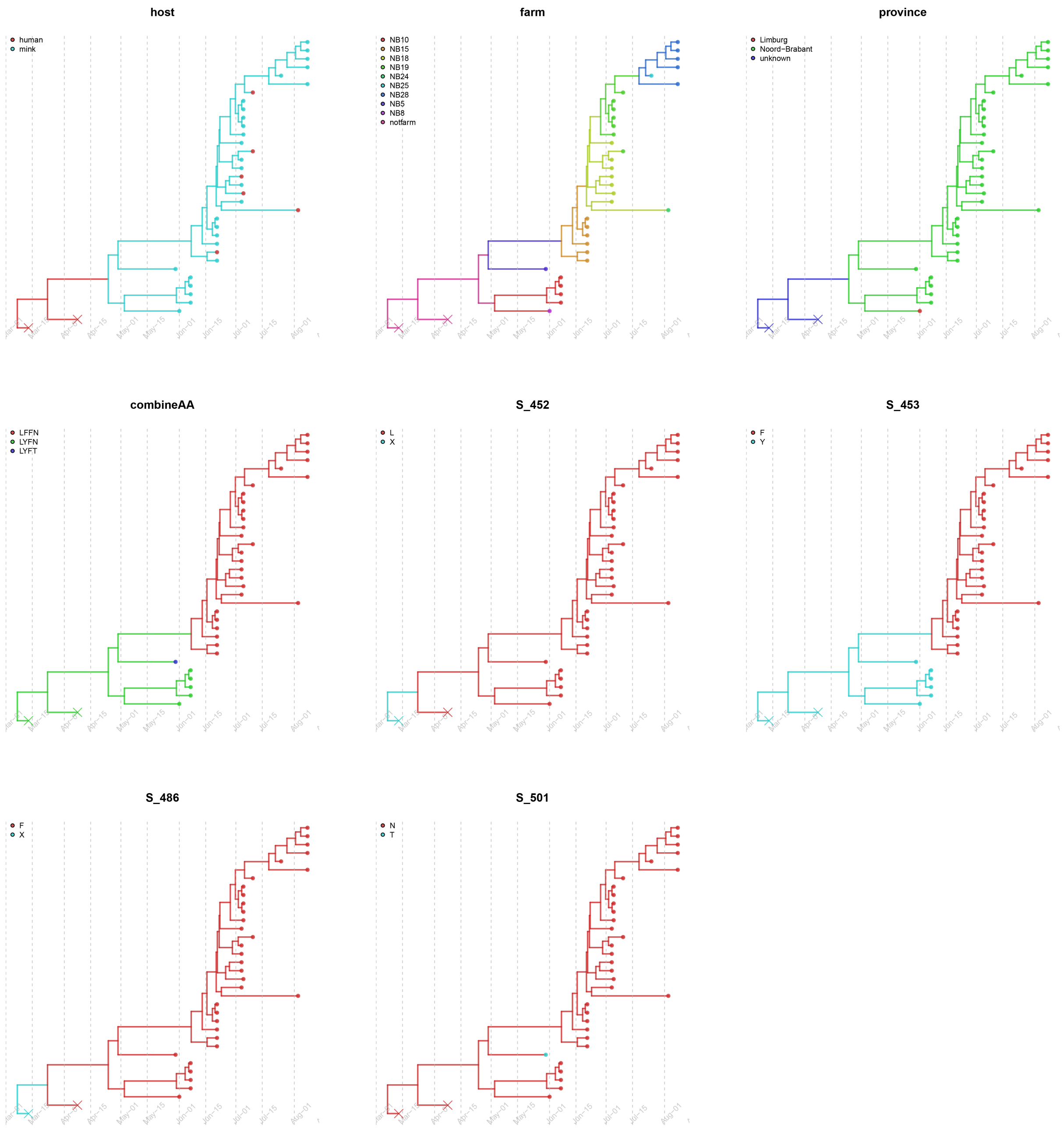

Figure S2. Discrete trait mapping on time-scaled phylogeny of Cluster D. Eight traits including host, farm ID (farm), province, the 4 unique sites in the spike protein with amino acid changes (L452M, Y453F,F486L and N501T) and the combinations of the 4 sites (combineAA) are mapped on the same MCC tree using the discrete trait model. The traits are plotted individually and for each tree, the branches and nodes are colored by inferred ancestral traits. Samples which fell within mink clusters but not isolated from farms are highlighted in diamond (1 escaped mink and 1un-associated human sequences). The outgroup containing human samples are cross labelled.

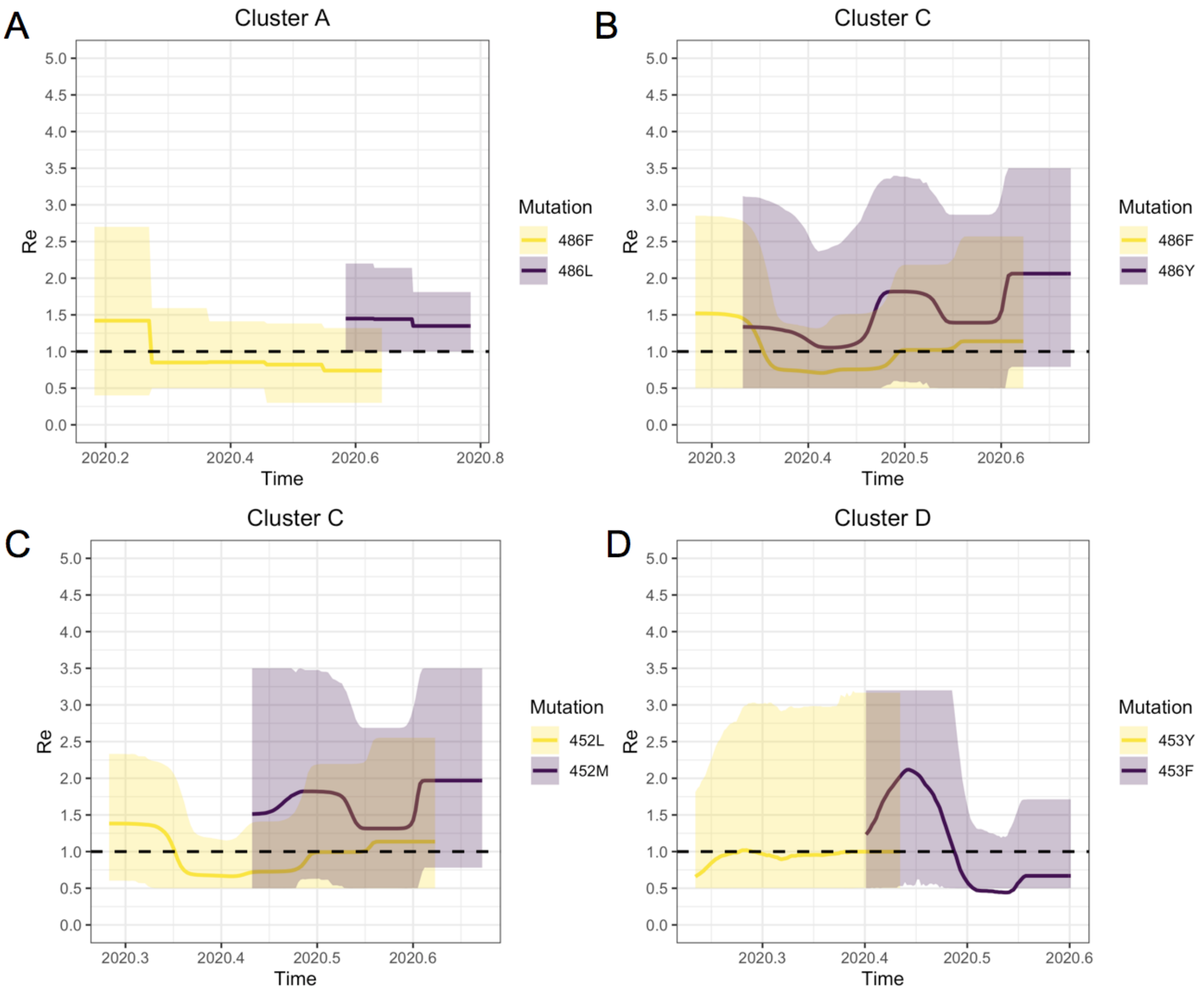

Figure S3 Multitype birth-death (BDMM) analysis of impact of appearance of mutations on Spike on reproductive number over time. For Cluster A, C and D, the changes in the *Re* for clades with amino acid changes on Spike protein site 486, 452 and 453 are shown in purple, clades without changes are shown in yellow. The shaded portion is the 95% HPD interval, and the solid line is the posterior median. Horizontal dashed line is *Re*=1.

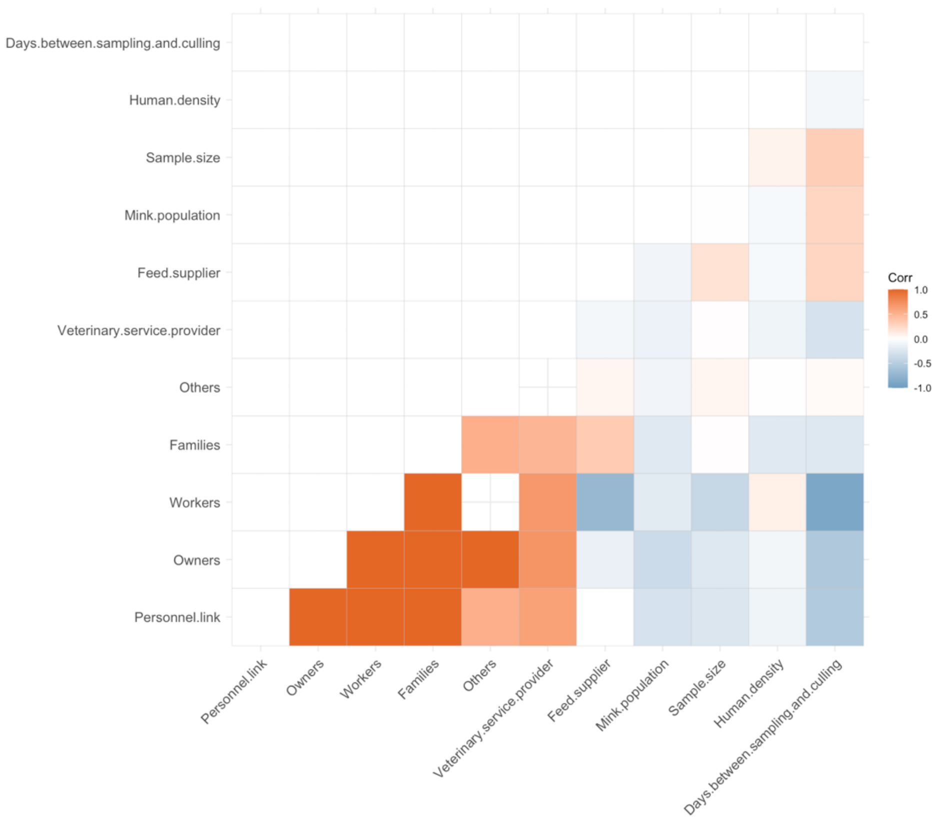

Figure S4 Correlation matrix between the predictor data collected from mink farms. The color corresponding to the correlation coefficient. Dark blue indicates strong positive correlation whereas dark red indicates strong negative correlation. Highly correlated predictors (sharing owners, sharing employees, owned by other members of the same family, or other links) were omitted.

Supporting files

Supplementary Data 1. SARS-Cov-2 sequences isolated from minks and humans used in the phylogenetic analysis. Metadata include Virus.name, GISAID Accession ID, Cluster number, Host, Farm ID, Collect Date, Amino acid substitutions in Spike protein.

Supplementary Data 2. The epidemiology metadata of mink farm NB1-68. Metadata include Farm ID, Epi-week, Mink cluster, Province, Human density (per sq km per town), Number of isolated sequences, Mink population, Days until final culling, Code for veterinary practice, feed company and personnel links.
