# Supplementary material for: Adaptation, spread and transmission of SARS-CoV-2 in farmed minks and associated humans in the Netherlands
Source: Nat Commun. 2021 Nov 23;12:6802. doi: 10.1038/s41467-021-27096-9 (PMC8611045; doi:10.1038/s41467-021-27096-9)
Supplement: Supplementary file 2 — Reporting summary. [file 41467_2021_27096_MOESM2_ESM.pdf]

nature portfolio

Corresponding author(s): Marion P. G. Koopmans

Last updated by author(s): Oct 29, 2021

Reporting Summary

Nature Portfolio wishes to improve the reproducibility of the work that we publish. This form provides structure for consistency and transparency in reporting. For further information on Nature Portfolio policies, see our Editorial Policies and the Editorial Policy Checklist.

Statistics

For all statistical analyses, confirm that the following items are present in the figure legend, table legend, main text, or Methods section.

n/a	Confirmed	
☐	☒	The exact sample size (*n*) for each experimental group/condition, given as a discrete number and unit of measurement
☐	☒	A statement on whether measurements were taken from distinct samples or whether the same sample was measured repeatedly
☐	☒	The statistical test(s) used AND whether they are one- or two-sided<br>*Only common tests should be described solely by name; describe more complex techniques in the Methods section.*
☐	☒	A description of all covariates tested
☒	☐	A description of any assumptions or corrections, such as tests of normality and adjustment for multiple comparisons
☐	☒	A full description of the statistical parameters including central tendency (e.g. means) or other basic estimates (e.g. regression coefficient) AND variation (e.g. standard deviation) or associated estimates of uncertainty (e.g. confidence intervals)
☒	☐	For null hypothesis testing, the test statistic (e.g. *F*, *t*, *r*) with confidence intervals, effect sizes, degrees of freedom and *P* value noted<br>*Give P values as exact values whenever suitable.*
☐	☒	For Bayesian analysis, information on the choice of priors and Markov chain Monte Carlo settings
☒	☐	For hierarchical and complex designs, identification of the appropriate level for tests and full reporting of outcomes
☒	☐	Estimates of effect sizes (e.g. Cohen's *d*, Pearson's *r*), indicating how they were calculated

Our web collection on statistics for biologists contains articles on many of the points above.

Software and code

Policy information about availability of computer code

Data collection	No software used for data collection.
Data analysis	minimap2 and python2 (pysam module): sequence trimming and mapping<br>MAFFT 7: sequence alignment<br>IQtree 2: Initial phylogenetic analyses<br>TempEst v1.5: measuring the root-to-tip divergence<br>BEAST 1.10.4 (skygrid, continuous model, discrete trait + GLM model) and BEAST 2.6.3 (package GEO-SPHERE, BDSKY and BDMM): Phylodynamic analyses<br>FigTree v1.4.3: tree visualizing<br>R 3.6.3 (packgae dplyr, ape and ggplot2): result data visualizing and manipulating

For manuscripts utilizing custom algorithms or software that are central to the research but not yet described in published literature, software must be made available to editors and reviewers. We strongly encourage code deposition in a community repository (e.g. GitHub). See the Nature Portfolio guidelines for submitting code & software for further information.

Data

Policy information about availability of data

All manuscripts must include a data availability statement. This statement should provide the following information, where applicable:

- Accession codes, unique identifiers, or web links for publicly available datasets
- A description of any restrictions on data availability
- For clinical datasets or third party data, please ensure that the statement adheres to our policy

The authors declare that the details of the data supporting the findings of this study are available within the article and in Supplementary Data files. The mink associated SARS-CoV-2 sequences generated in this study have been deposited into the European Nucleotide Archive (ENA) (https://www.ebi.ac.uk/ena/browser/home), and the accession IDs are listed in the Supplementary Data 1. The source data including metadata of all the sequences and epidemiology data used in all analyses are available in Supplementary Data 1 and 2， respectively.

Field-specific reporting

Please select the one below that is the best fit for your research. If you are not sure, read the appropriate sections before making your selection.

☐ Life sciences ☐ Behavioural & social sciences ☒ Ecological, evolutionary & environmental sciences

For a reference copy of the document with all sections, see nature.com/documents/nr-reporting-summary-flat.pdf

Ecological, evolutionary & environmental sciences study design

All studies must disclose on these points even when the disclosure is negative.

Study description

We described the course of SARS-CoV-2 outbreaks in minks and related humans in mink farms in the Netherlands and explored how SARS-CoV-2 in minks and related humans on farms was transmitted by combining sequence data and epidemiological information with Bayesian phylodynamic methods. Three different clusters were analysed independently and variations of the phylodynamic lineage growth rate during the time of assessment (spanning several months in total) were observed, and a trend towards increased growth rates coinciding with the appearance of clades with spike protein mutations. Directions and number of transmissions between hosts and farms were quantified by discrete trait models. Furthermore, the contribution of a range of predictor variables to the spread of viruses between farms was estimated and the movement of people and distance between farms were determined as statistically significant predictors of virus dispersal between farms.

Research sample

Full virus genome sequences, including location information and sampling date, of 295 minks from 64 out of the 68 mink farms in the Netherlands were obtained; 218 sequences isolated from randomly selected patients from 31 postal codes in the region of SARS-CoV-2 positive mink farms were obtained to assess possible spill-over to the local community. In addition, all sequences submitted to GISAID from the Netherlands until the 4th of January were subsampled and included in the analysis; all available GISAID sequences from the Netherlands were checked for mink associated strains.

Moreover, a selection of metadata, on farm-farm contacts (mink, humans, veterinarian, feed company) were assessed for their possible role in farm-to-farm transmission.
Mink samples were taken from kits,female adults and some male adults and were selected based on likeliness of being positive (either by showing clinical signs or being present in/near cages nearby previously shown increased mortality or clinical signs). Kits were born half April/May, so ages ranged from a few weeks to 7 months of age. Adults were born in the preceding years (50% in 2019, 30% of 2018 and 20% in 2017). Kits were 50/50 males and females. Most adults were females. Age distribution on the farm was generally 84,5% kits, 15% females and 0,5% males. The human samples were from the farm workers and their families which included males and females and children and young, medium-aged and senior adults.

Sampling strategy

Per farm, a maximum of five samples with the lowest Ct-value (but at least below Ct 32) were subjected to WGS, as well as all known human farm contacts with Ct value <32. Complete SARS-CoV-2 genomes isolated from minks and related humans were included in the phylodynamic reconstructions. To avoid sample size effect on the results, sequences were further subsampled to reduce over-representative sequences from the same farm. We also included human sequences from across the Netherlands as background data, which being subsampled to keep at least 1 sequence per global lineage as defined using the Pango-lineage classification (version 1st April 2021) per region per week.

Data collection

Mink farms suspected of SARS-CoV-2 infections were visited for sampling and epidemiological investigation by the competent authority (Netherlands Food and Consumer Product Safety Authority, NVWA). Official sampling included non-random sampling of 20 minks, by means of throat and rectal swabs, targeting minks with clinical signs. Data collected for each farm included farm location, number of animals, ownership, shared personnel and other contacts (anonymised), data of confirmed SARS-CoV-2 detection and time interval between sampling and culling.

On the first SARS-CoV-2 infected mink farms, NB1-NB16 (NB is the Dutch abbreviation for mink farm, which were numbered consecutively based on diagnosis) active case finding as well as serum collection of people with possible exposure to infected minks was performed. On farms NB17-NB68, all owners and employees of infected mink farms were requested to visit a regional SARS-CoV-2 testing facility in case of any symptoms indicative of COVID-19, in line with the national SARS-CoV-2 testing and surveillance policy.

Timing and spatial scale

Between 24th April 2020 to 4th November 2020, full genome sequences of 295 minks from 64 out of the 68 mink farms in the

	Netherlands were obtained; two screenings of SARS-CoV-2 positive humans living in the same region as the infected mink farms took place from 3rd April 2020 to the 4th of Junuary 2020; SARS-CoV-2 sequences from humans across the Netherlands were randomly selected and the collected date was up to 4th January 2021.
Data exclusions	Complete SARS-CoV-2 genomes with >95% coverage isolated from minks and related humans were included in the phylodynamic reconstructions. SARS-CoV-2 sequences from humans across the Netherlands were selected and the collected date was up to 4th January 2021. Overrepresentative sequences were excluded by randomly selected one unique genome per region, epiweek and pango-lineage.
Reproducibility	The authors confirmed that all analyses can be reproduced successfully from the raw data using the methodology described in the manuscript.
Randomization	Results of transmission patterns and impact of predictors did not vary using data from repeated subsampling sequences within the same trait groups.
Blinding	Investigators were not blinded to group allocation during data collection and analysis. And blinding is not relevant as this study is not experimental.

Did the study involve field work? ☒ Yes ☐ No

Field work, collection and transport

Field conditions	Human and animal swabs were cooled immediately after collection and transported to the laboratory. PCR testing was executed within 3 days. Whole genome sequencing was done after cooled transport to the ErasmusMC. Most samples experienced an additional freeze-thaw cycle before sequencing.
Location	Sampling was on on-farm (minks; locations in manuscript) or at the nearest COVID-19 testing location (humans).
Access & import/export	All samples stayed within the Netherlands, no permits necessary.
Disturbance	Samples were collected as part of the national COVID-19 response and control efforts. No additional disturbance caused by this study.

Reporting for specific materials, systems and methods

We require information from authors about some types of materials, experimental systems and methods used in many studies. Here, indicate whether each material, system or method listed is relevant to your study. If you are not sure if a list item applies to your research, read the appropriate section before selecting a response.

Materials & experimental systems

n/a	Involved in the study	
☒	☐	Antibodies
☒	☐	Eukaryotic cell lines
☒	☐	Palaeontology and archaeology
☐	☒	Animals and other organisms
☐	☒	Human research participants
☒	☐	Clinical data
☒	☐	Dual use research of concern

Methods

n/a	Involved in the study	
☒	☐	ChIP-seq
☒	☐	Flow cytometry
☒	☐	MRI-based neuroimaging

Animals and other organisms

Policy information about studies involving animals; ARRIVE guidelines recommended for reporting animal research

Laboratory animals	none
Wild animals	none
Field-collected samples	Samples collected from farmed mink, kept under national animal welfare rules and according to the standards as specified in Welfur standards: https://www.sustainablefur.com/wp-content/uploads/2018/11/Mink_protocol_final_web_edition_light.pdf
Ethics oversight	No ethical permission were necessary since all sample had been collected under national covid-19 response and control efforts and regulations.

Note that full information on the approval of the study protocol must also be provided in the manuscript.

Human research participants

Policy information about studies involving human research participants

Population characteristics	All samples were collected as part of COVID-19 control policy, and processed anonimously.
Recruitment	Mandatory (national policy) sampling in case of COVID-19 complaints or COVID-19 contact. Some human who were mink farm contacts were tested voluntarily while asymptomatic.
Ethics oversight	Outbreak investigations of notifiable diseases such as COVID-19 are the legal tasks of the Public Health Service as described under the Public Health Act, and do not require separate medical ethical clearance.

Note that full information on the approval of the study protocol must also be provided in the manuscript.
