# Supplementary material for: Adaptation, spread and transmission of SARS-CoV-2 in farmed minks and associated humans in the Netherlands
Source: Nat Commun. 2021 Nov 23;12:6802. doi: 10.1038/s41467-021-27096-9 (PMC8611045; doi:10.1038/s41467-021-27096-9)
Supplement: Supplementary file 3 — Description of Additional Supplementary Files. [file 41467_2021_27096_MOESM3_ESM.pdf]

Description of Additional Supplementary Files

Supplementary Data 1. SARS-Cov-2 sequences isolated from minks and humans used in the phylogenetic analysis. Metadata include Virus.name, ENA and GISAID Accession ID, Cluster number, Host, Farm ID, Collect Date, Amino acid substitutions in Spike protein.

Supplementary Data 2. The epidemiology metadata of mink farm NB1-68. Metadata include Farm ID, Epi-week, Mink cluster, Province, Human density (per sq km per town), Number of isolated sequences, Mink population, Days until final culling, Code for veterinary practice, feed company and personnel links.
